# The Influence of Light and Physical Activity on the Timing and Duration of Sleep: Insights from a Natural Model of Dance Training in Shifts

**DOI:** 10.3390/clockssleep5010006

**Published:** 2023-01-31

**Authors:** Ignacio Estevan, Natalia Coirolo, Bettina Tassino, Ana Silva

**Affiliations:** 1 Grupo Cronobiología, Comisión Sectorial de Investigación Científica, Universidad de la República, Montevideo 11300, Uruguay; 2 Programa de Neuropsicología y Neurobiología, Facultad de Psicología, Universidad de la República, Montevideo 11300, Uruguay; 3 Sección Etología, Facultad de Ciencias, Universidad de la República, Montevideo 11400, Uruguay; 4 Laboratorio de Neurociencias, Facultad de Ciencias, Universidad de la República, Montevideo 11400, Uruguay

**Keywords:** accelerometry, educational shifts, illuminance, physical activity, sleep

## Abstract

Environmental, social, and behavioral variables influence sleep timing and duration. Using wrist-worn accelerometers, we recorded 31 dancers (age = 22.6 ± 3.5) for 17 days and who trained either in the morning (*n* = 15) or in the late evening (*n* = 16). We estimated the dancers’ daily sleep pattern: onset, end, and duration. In addition, their minutes of moderate-to-vigorous physical activity (MVPA) and mean light illuminance were also calculated daily and for the morning-shift and late-evening-shift time windows. On training days, the shifts involved differences in sleep timing, alarm-driven waking frequency, and the pattern of light exposure and MVPA duration. Sleep was strongly advanced when dancers trained in the morning and when alarms were used, while morning light had a low influence. Sleep was delayed when dancers were more exposed to light and displayed longer MVPA during the late evening. Sleep duration was strongly reduced on weekends and when alarms were used. A small reduction in sleep duration was also observed when morning illuminance was lower or when late evening MVPA was longer. Training in shifts influenced the timing of environmental and behavioral factors, which added up to shape dancers’ sleep timing and duration.

## 1. Introduction

Sleep characteristics, both quantitative and qualitative, are shaped by environmental, social, and behavioral influences. Impaired sleep timing, duration, and quality have major consequences on health as well as on mental and physical performance [1]. Social pressures are relevant factors affecting sleep in urban life. For example, late-oriented individuals suffer from sleep restrictions on weekdays because of early social duties and compensate for this sleep deprivation by oversleeping on weekends [2]. For teenagers, who have extreme nocturnal preferences and chronic sleep deprivation, the social demand of an early school start time is a major challenge for their sleep [3]. Although college students are not as late oriented as adolescents, 40% of them still report sleep durations of less than 6 h per day [4]. The prevalent use of alarms on weekdays by students is an indicator of the high level of youngsters’ misalignment between their internal and external clocks [5].

Sleep is partially controlled by the circadian system, which has ambient light as its main entrainer [6]. The increase in artificial light exposure during the night and the decrease in natural light exposure during the day are involved in the delayed onset of daily sleep and in sleep deficits [6,7]. Some field studies show that low daily light exposure and high artificial light during the evening are associated with delayed sleep [8,9,10]. In contrast, the evidence regarding the effect of light on sleep duration is contradictory and requires further validation [9,11,12,13].

Physical activity and sleep influence each other [14]. Both chronic and acute daytime physical activity have been associated with longer sleep durations [15]. Physical activity can entrain the circadian system to act independently of as well as additively regarding the effect of light [16,17]. Programmed exercise can, thus, advance the circadian phase and, secondarily, sleep if practiced in the morning or delay them if practiced during the evening [18]. Even though avoiding evening exercise is recommended to have a good subsequent sleep episode [19], recent reviews found no significant support for the negative association between late exercise and sleep [20,21].

School shifts are advantageous models to study how the interactions of social pressures and the circadian system influence adolescent sleep [22,23,24]. Sleep occurs earlier and shorter in morning-shift high school students compared to afternoon-shift students [22,23]. As shown in shift workers [25,26], school shifts also imply differences in light exposure [24] that can be associated with differential sleep and activity patterns. However, no differences in physical activity associated with school shifts have been found so far [23]. Overall, it is not yet completely clear what school shifts entail in terms of social and/or circadian challenges.

Uruguayan youngsters have been described as extremely late oriented with a strong misalignment and chronic sleep deficit [22,27,28]. Daily illuminance induces an early and longer sleep in Uruguayan adolescents, but this effect is not enough to counteract the sleep deficit produced by early school start times [12]. Uruguayan public school for professional training in dance operates on two separate shifts that strongly influence dancers’ self-reported sleep patterns, with the sleep of late-evening-shift dancers being delayed relative to that of the morning-shift dancers during training days [27]. In this study, we first aimed to objectively characterize the sleep patterns of morning-shift and late-evening-shift dancers. We then evaluated the influence of dance-training shifts on the physical environment (light exposure), dancers’ behavior (physical activity), and forced awakening (the use of alarm clocks). We finally took advantage of this natural experiment to study the unique and additive influences of these factors on dancers’ sleep patterns.

## 2. Results

We studied the sleep pattern of 31 college dance students who trained either on a morning shift (23.1 ± 2.9 years old) or on a late evening shift (22.2 ± 4.0 years old), with age not being different between shifts (*t* (29) = −0.7, *p* = 0.523). An average of 15.6 ± 1.0 daily records per participant were considered (range between 11 and 16), which consisted of 11.7 ± 0.7 sleep episodes before a training day (Monday through Friday; range between 9 and 12) and 3.8 ± 0.5 sleep episodes before a weekend day (Saturday and Sunday; range between 2 and 4).

### 2.1. Sleep Timing between Shifts and Type of Day

**Figure 1:**
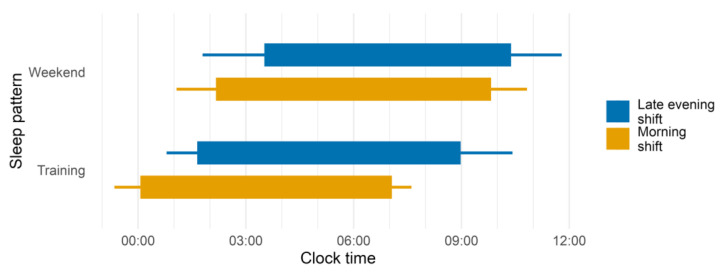
Sleep pattern by type of day and training shift. Sleep schedule on weekend days and training days in dancers attending either the morning (orange bars, 08:30 to 12:30, *n* = 15) or the late evening training shift (blue bars, 20:00 to 24:00, *n* = 16). Each rectangle extends from the grand mean sleep onset to the grand mean sleep end. Lines represent standard deviation.

As shown in Figure 1 and Table 1, sleep onset was delayed in late-evening-shift dancers compared to morning-shift ones (b = 1.4 ± 0.3, *p* < 0.001) and on weekend vs. training days (b = 2.0 ± 0.1, *p* < 0.001), with no significant interaction (pseudo-R2 marginal/conditional = 0.37/0.52). Regarding sleep end, the interaction between training shift and type of day was significant (pseudo-R2 marginal/conditional = 0.37/0.59). We found that it was earlier in training days among dancers of both shifts compared to weekend days (morning shift: b = −2.8 ± 0.2, *p* < 0.001; late evening shift: b = −1.4 ± 0.2, *p* < 0.001), and earlier among morning-shift dancers compared to late-evening-shift ones only on training days (b = −1.9 ± 0.4, *p* < 0.001). In relation to sleep duration, the interaction was significant, and only morning-shift dancers slept longer on weekend days than on training days (b = −0.6 ± 0.2, *p* = 0.036; pseudo-R2 marginal/conditional = 0.02/0.11).

### 2.2. Training Shifts Involve Differences in the Timing of Light Exposure and Physical Activity

Training shifts entail differences between training days and weekend days as well as differences between shifts (Table 2). There was a significant interaction between the type of day and the training shift for daily MVPA, which did not differ between shifts during training days. However, during weekend days, morning-shift students spent nearly 40 min less in MVPA than late-evening-shift students (*p* = 0.011). In addition, the time spent in MVPA was less on weekend days than on training days for morning-shift dancers (*p* < 0.001). Additionally, significant interactions between the type of day and training shift were also found when morning and late evening MVPA were considered (Table 2). On training days, morning-shift dancers exhibited longer MVPA than late-evening-shift dancers during the morning, as expected (*p* < 0.001) and when compared to their own weekend mornings (*p* < 0.001). An inverse pattern was observed during the late evening, when late-evening-shift dancers exhibited longer MVPA than morning-shift ones (*p* < 0.001) and when compared to their own late evenings (*p* < 0.001).

Daily illuminance was similar between the type of days and training shifts. On the other hand, higher illuminance was observed for training vs. weekend days during the morning (exponentiated mean log_10_ values were 232.0 ± 3.0 vs. 76.0 ± 6.6 lux). Only on training days was the late evening illuminance higher for late-evening-shift dancers than in morning-shift ones (*p* < 0.001). In addition, only for late-evening-shift dancers was the illuminance also higher on training days than on weekend days (*p* < 0.001).

Dancers used an alarm clock to wake up for 70.7% of the total observations (Table 2). The proportion of alarm usage was higher on training vs. weekend days for both shifts (morning shift: *p* < 0.001; late evening shift: *p* < 0.001), with this difference being even higher for morning-shift dancers than for late-evening-shift dancers. In addition, the proportion of alarm usage was higher for morning-shift dancers than for late-evening-shift ones, although only on training days (*p* < 0.001).

### 2.3. Sleep Pattern Is Influenced by Several Factors Related with Training in Different Shifts

**Figure A1:**
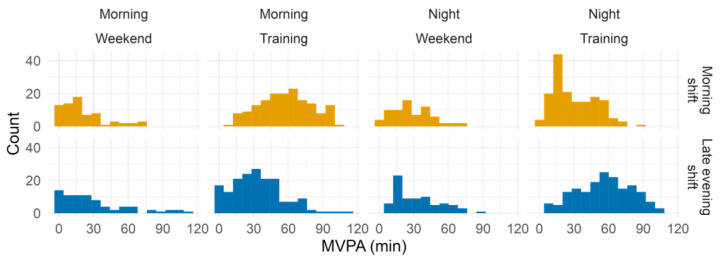
Moderate-to-vigorous activity distribution. Histograms showing the minutes of moderate-to-vigorous physical activity distribution (*n* = 31; 461 observations in the morning window and 476 observations in the late evening window) by type of day (training/weekend), time window (morning: 08:30 to 12:29; late evening: 20:00 to 23:59), and training shift (morning: 08:30 to 12:29; late evening: 20:00 to 23:59).

**Figure A2:**
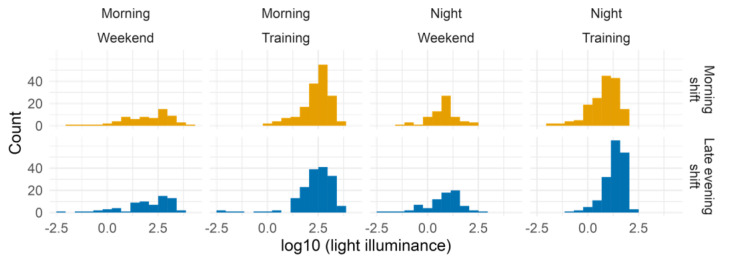
Illuminance distribution. Histograms showing the illuminance distribution (*n* = 31; 483 observations in the morning window and 483 observations in the late evening window) by type of day (training/weekend), time window (morning: 08:30 to 12:29: late evening: 20:00 to 23:59; 483 observations in the morning), and training shift (morning: 08:30 to 12:29: late evening: 20:00 to 23:59; 483 observations in the morning).

As shown in Figure A1 and Figure A2, there is great variation both between and within shifts and types of day in illuminance and in the duration of MVPA. We used this variation as well as the frequency of alarm usage, the type of day, and the training shift to evaluate the influence of each of these variables on sleep patterns. As shown in Table 3, sleep onset was strongly influenced by both the training shift (b = 68 ± 16 min delay for late-evening-shift dancers, *p* < 0.001) and the type of day (b = 113 ± 9 min delay on weekend days, *p* < 0.001). No influence by the use of alarm clocks on sleep onset was found. The log_10_ daily illuminance was associated with an advanced sleep onset (non-standardized b = −15 ± 7 min, *p* = 0.027; Table A1). When illuminance was analyzed separately in the morning and in the late evening time windows, a 10-fold increase in morning illuminance was associated with a 20 ± 4 min advance in sleep onset (*p* < 0.001), while the same increase in late evening illuminance was associated with a 12 ± 6 min delay (*p* = 0.036). On the other hand, daily MVPA was not associated with changes in sleep onset (Table A1). However, a 10 min increase in the late evening time window MVPA was associated with a 9 ± 2 min delay in sleep onset (*p* < 0.001). When previous sleep end was included as control, it appeared as a significant predictor, while only previous late evening illuminance became non-significant (Table A2).

Training shift, type of day, and alarm usage were the strongest predictors of sleep end. Late-evening-shift dancers woke up 75 ± 22 min later than morning-shift ones (*p* = 0.002). On weekend days, their sleep end was 70 ± 9 min later (*p* < 0.001). Alarm usage was associated with a 97 ± 9 min earlier sleep end (*p* < 0.001). In addition, log_10_ daily illuminance was associated with an advanced sleep end (non-standardized b = −19 ± 6 min, *p* = 0.002; Table A1), while a 10-fold increase in morning illuminance was associated with an 8 ± 4 min advance in sleep end (*p* = 0.038); a similar increase in late evening illuminance was associated with a 14 ± 5 min delay (*p* = 0.007). Time spent daily on MVPA was not associated with sleep end for either the morning or the late evening. Previous sleep end was not a significant predictor but influenced morning illuminance to become non-significant (Table A2).

Overall, the changes in sleep onset and sleep end explain the observed strong association of sleep duration with the type of day and alarm usage. Sleep duration was reduced by 43 ± 10 min on weekend days compared to training days (*p* < 0.001), and alarm usage *per se* reduced it by 99 ± 10 min (*p* < 0.001). In addition, morning illuminance was associated with an increase in sleep duration (non-standardized b = 10 ± 5 min, *p* = 0.024), while a 10 min increase in late evening MVPA was associated with a shorter sleep duration (non-standardized b = −7 ± 2 min, *p* = 0.001). Inclusion of the previous sleep end did not modify the described associations (Table A2).

## 3. Discussion

Using dance training in shifts as an advantageous field model system, we showed, for the first time in a single study, the interplay of the environmental, social, and behavioral variables influencing sleep timing and duration in students. Previous reports showed how social pressures such as school shifts are associated with differences in sleep patterns between shifts and between workdays and weekends [22,27,29]. In this study, we first described the profound differences that dance training in shifts implies not only for sleep patterns, but also for light exposure, physical activity, and sleep constraints (forced awakening). We also explored the contribution of each of these factors to sleep patterns and duration. In addition to the expected impact by the different shifts, we found the following: (a) a strong influence by forced awakening on advancing sleep and reducing its duration; (b) a slight influence by morning light exposure on advancing sleep onset and increasing its duration; (c) a moderate influence by late evening light exposure on delaying sleep; and (d) a slight influence by late evening intense exercise on delaying sleep onset and decreasing its duration.

### 3.1. What Do Dance-Training Shifts Entail?

Dance training in shifts constitutes a strong and complex social pressure. While there were no differences in daily mean illuminance between shifts on training days, light was higher on training days compared to weekend days for dancers of both shifts. Similar results were found between work and weekend days for adults [30,31] and in school vs. weekend days for adolescents [12]. Morning illuminance was also higher on training days than on weekends, which is likely related with the increased exposure to natural light that training days imply. In addition, as previously reported [24,32], morning-shift dancers were more exposed to light in the morning, while late-evening-shift dancers were more exposed to light in the late evening. However, a significant interaction was found for late evening illuminance, which was higher in late-evening-shift dancers compared to both their morning light exposure and to the morning-shift dancers’ late evening light exposure.

Regarding physical activity, the time spent on MVPA was, as expected, well above the time recommended for this age group [33], which was similar for dancers of both shifts on training days and longer during the time window that matched the dancers’ training shift. A previous study in adolescents also found no differences between shifts for students who did not receive specific physical training [23].

We conceived the use of alarms as a proxy for the impact of training shifts as social pressure. As previously reported in other populations of students [5], their use was prevalent on training days, particularly among morning-shift dancers. Interestingly, forced awakening was less prevalent but still frequent in late-evening-shift dancers and during weekends, suggesting that other pressures than those that are training-shift-related restricted the dancers’ sleep.

### 3.2. What Factors Shape the Sleep Pattern?

In this study, we addressed how training shifts, and the other variables that training in shifts entails, influence sleep patterns. When all the variables were considered together (training shift, type of day, alarm use, light exposure, and physical activity), we confirmed that sleep timing but not sleep duration differed between shifts. In addition, this comprehensive analysis identified a significant influence by the other variables (type of day, alarm use, light exposure, and physical activity) on sleep timing and duration. Although these variables were associated with training shifts, not all of the influence of shifts on sleep was mediated by them.

As expected, on weekend days, we found that sleep timing was delayed even after controlling for several other variables, with sleep onset delayed twice as long as sleep end [2]. On training days, mean sleep durations were above the recommended 7 h for this age group [34], which means that participants did not have a sleep debt to pay down on weekend days. Indeed, only in morning-shift dancers was sleep duration longer on weekend days. Interestingly, sleep duration (when controlling for the other variables) was reduced in the entire sample by almost 45 min on weekend days. Participants used wake-up alarms frequently and had similar levels of illuminance and MVPA on weekend days compared to training days, suggesting that their delayed sleep pattern could be colliding with other activities and pressures that we did not consider. In line with this, studies on school shifts in adolescents found that only morning-shift students showed reduced sleep durations on school days that they were unable to counteract by oversleeping on weekends [12,22,29]. We, thus, understand that weekend days are not that free, and that sleep is socially demanded by scheduled activities also on weekend days. This should be considered when studying sleep and making recommendations for sleep health improvement in youngsters.

Alarm clock usage was associated with an almost 2 h advanced sleep end and a similar reduction in sleep duration, which proved to be its strongest predictor. We found no association between the use of wake-up alarms and sleep onset. This suggests that it is difficult for young people to plan their activities to obtain adequate sleep durations; i.e., dancers seem unable to set their sleep onset according to the next day’s schedule.

Daily mean illuminance was associated with an advanced and longer sleep, as also observed in Uruguayan adolescents [12] and in a few previous reports using objective measures [11,13]. When segmented into the morning and the late evening time windows, illuminance had an opposite effect on sleep timing. As expected, based on laboratory studies of phase curve responses [35], higher illuminance in the morning advanced sleep onset and increased sleep duration, while higher illuminance during the late evening delayed both sleep onset and sleep end, with no effects on sleep duration. The dim-light salivary melatonin concentrations curve that was studied in a small group partially overlapped with that of the participants of this study, showing an advanced onset among morning-shift dancers compared to late-evening-shift ones [36], which can be related with the higher morning illuminance we found among the morning-shift students compared to the late-evening-shift ones.

Despite the previously reported positive influence of daily MVPA on sleep duration [15,37], we failed to find associations between the intensity of daily physical activity and either sleep duration or timing. Our negative results likely depended on the specific characteristics of our study population, who were good sleepers (sleep duration > 7 h for the weekly average) in general and practiced high levels of physical activity regularly because of their training. Therefore, there might be no room for dancers’ sleep improvements that are associated with variations in MVPA. However, we did find that late evening MVPA was associated with a delayed sleep onset and a reduced duration. It was shown that nocturnal physical activity increases pre-sleep physiological arousal (e.g., heart rate, [21]), which was related with poorer sleep and insomnia [38]. Furthermore, physical activity can also serve as an entrainer of the circadian system, delaying its phase angle by acting during the late evening [18], as has been observed in a previous report on a subsample of this study population of dancers [36]. This result adds evidence to a matter under current debate, for which previous studies have not reached a consensus. While some studies evaluating the influence of late evening exercise on sleep timing and duration found no effect [20,21], other reports showed an increase in sleep latency associated with nighttime exercise [39,40]. More comprehensive field studies are needed to understand the tradeoff between the benefits and harm of physical activity at night.

Our study had the costs and benefits of being a field study on a very specific population, in which the influential variables on sleep were not controlled at all. Although repeated measures increased the number of observations and enhanced the power of the statistical analysis, using a greater number of participants would have allowed us to reach more conclusive results and to deepen the interactions between explanatory variables. A greater number of observations on weekend days would also have been necessary. Furthermore, it would be interesting to calculate the phase estimators of the circadian system, such as chronotypes, and to study whether they interact with the factors shown here to influence the sleep pattern. It would have also been important to know more about the social pressures that are present on the weekend days, which forced dancers to use alarms and also influenced the patterns of light exposure and physical activity. In addition, the female bias of the sample prevented us from studying gender-related differences. Finally, it is important to note that despite the instructions to the participants to keep their accelerometers exposed, we cannot rule out that the light meter may have been covered by their sleeves, so the exposure may have been underestimated. Furthermore, Geneactiv devices are poor at estimating low illuminances [41], while underestimating high illuminances [42]. Moreover, more accurate recordings of spectral composition or melanopic illuminance at the gaze level would have allowed a better measurement of the light stimulus and, thus, allowed us to discuss if the light illuminance among participants is in line with the current recommendations [43].

## 4. Materials and Methods

The sleep patterns of 31 dancers (5 males, 1 non-binary) attending the Uruguayan public school for professional training in contemporary and folkloric dance (Escuelas de Formación Artística, END-SODRE, Ministerio de Educación y Cultura, Uruguay) were assessed from 11 August 00:00 to 27 August 23:59, 2019 (winter in the Southern Hemisphere) using wrist-worn GENEactiv Original accelerometers (Activinsights Ltd., Cambridge, UK). Participants were instructed to keep the devices continuously exposed to ensure the recording of their light exposure. Classes in the END-SODRE 4-year training program are taught annually from March to November and from Monday through Friday. Classes are scheduled in the late evening (20:00 to 24:00) for first- and second-grade students and in the morning (08:30 to 12:30) for third- and fourth-grade students [27,36]. The END-SODRE curriculum involves practical training for at least three hours per day. Participants aged between 18 and 34 (22.6 ± 3.5) were either trained on the morning shift (*n* = 15) or on the late evening shift (*n* = 16).

Accelerometer and light exposure data were acquired using a 10 Hz sampling frequency, as described elsewhere [36]. Daily sleep onset and sleep end were estimated from accelerometry recordings using GGIR library [44], and sleep-logs guided the automatic classification of sustained inactive periods as night sleep. During the study period, participants also completed a digital daily sleep log, in which they reported their previous night sleep onset and sleep end as well as their usage of an alarm clock to wake up. Sleep duration was calculated as the difference between sleep onset and sleep end, resulting in 483 sleep episodes to be considered after the exclusion of incomplete data (approximately 2%). The lack of information in the sleep diaries led to the exclusion of 6 observations. The removal of the devices or their malfunctioning led to the exclusion of another 7 observations.

Physical activity was estimated using an adaptation of GGIR library [45,46]. Using Euclidean Norm Minus One (ENMO) with negative values set to zero multiplied by 1000, minutes of moderate-to-vigorous physical activity (MVPA) were estimated. We used ENMO > 92 mg as the threshold for MVPA [47]. Light exposure was estimated as illuminance (lux) from wrist-worn accelerometers recordings. Individual recordings were converted into 1 min bins and segmented from 00:00 to 23:59. For both variables, mean values were calculated by day and for each of the respective shifts: morning (08:30 to 12:29) or late evening (20:00 to 23:59). In the case of light, we calculated the log_10_ of the mean light illuminance for each time window, and 29 values with zero mean illuminance were discarded.

We used mixed-effects linear regressions with ID as random effect to study differences in daily, morning, and late evening physical activity, considering the training shift and the type of day as predictors. A generalized linear model was employed to study differences in the frequency of alarm usage by shift. We used mixed-effects linear regressions with id as random effect to explain day-to-day variance in the sleep behavior. Training shift (morning/late evening), next day type of day (training/weekend), use of an alarm clock (yes/no), and previous day light exposure and physical activity were considered as predictors. The temporal relationship of the predictor variables included with their corresponding sleep episode allowed us to study their influence on sleep and to assign a direction to the association found in this correlational analysis. Time was converted to decimal hours, and continuous predictors were mean-centered and standardized. Degrees of freedom were calculated via Satterthwaite’s method using lmerTest [48]. Bonferroni correction was employed to adjust for multiple comparisons. Calculation of marginal and conditional pseudo-R2 were computed using MuMIn [49]. Residuals were plotted and inspected for deviations in normality or homoscedasticity.

Descriptive statistics are presented as mean ± standard deviation or frequency (percentage). For the regressions, the marginal/conditional pseudo-R2 and the standardized coefficients (b/z) ± standard error with the associated *p*-values are reported.

## 5. Conclusions

The real-life model system of dancers being trained in two extreme shifts involved differences across shifts in the sleep timing, alarm-driven waking frequency, light exposure pattern, and pattern of intense physical activity. Continuous accelerometer recordings generated repeated observations of these variables of interest, along with an organization of the database centered on each sleep episode. We were thus able to study how each observation of each variable acted on each sleep episode. In this scenario, we found that sleep was strongly advanced when dancers were trained in the morning the day before and when alarms were used the next morning, while previous morning light had a significant but low influence. Furthermore, subsequent sleep was delayed when dancers were exposed to higher illuminances and displayed longer MVPA during the late evening. Finally, sleep duration was strongly reduced before a weekend day and when alarms were used the next morning, while lower previous morning illuminance or longer previous late evening MVPA generated a small reduction in the next sleep episode duration.

This field model system also allowed us to carry out a comprehensive analysis of the complex interplay of the social, environmental, and behavioral variables influencing the sleep patterns in a single study. In other words, the social pressure of shift-training imposed differences on the timing of both environmental factors and behavioral displays, with an interplay that influenced sleep patterns in a distinctive and complex manner. For example, (a) morning light exposure, but not morning physical activity, advanced sleep and increased its duration; (b) evening light exposure and physical activity delayed sleep, but only evening intense physical activity reduced sleep duration; and (c) the unexpected sleep reduction associated with weekend days was, partially or totally, overcome by a lower use of alarms on these days. This field study, thus, demonstrated how relevant and tricky these diverse variables can be in shaping the natural sleep pattern, revealing complex associations that remain underestimated in laboratory-controlled conditions.

## Figures and Tables

**Table 1:** Sleep pattern grand mean values by type of day and training shift. Sleep schedule on weekend days and training days in students attending either the morning (08:30 to 12:30, *n* = 15) or the late evening training shift (20:00 to 24:00, *n* = 16).

Sleep	Morning Shift		Late Evening Shift	
	Training Day	Weekend Day	Training Day	Weekend Day
Onset (h)	00:04 ± 00:43	02:10 ± 01:06	01:39 ± 00:51	03:31 ± 01:43
End (h)	07:04 ± 00:33	09:49 ± 01:00	08:59 ± 01:27	10:23 ± 01:25
Duration	7.0 ± 0.6	7.7 ± 0.8	7.3 ± 0.9	6.9 ± 1.2

**Table 2:** Alarm usage, light exposure, and physical activity in morning and late evening shifts, on training and weekend days.

	Time Window	Morning Shift (*n* = 15)		Late Evening Shift (*n* = 16)		Training Shift	Type of Day	Training Shift * Type of Day
		Training Day	Weekend Day	Training Day	Weekend Day			
Moderate-to-vigorous physical activity (MVPA, minutes) ^1^	Daily	184.5 ± 40.0	143.7 ± 36.9	182.6 ± 37.2	190.7 ± 57.6	F(1,30.1) = 2.6, *p* = 0.114	F(1,450.3) = 11.3, *p* < 0.001	F(1,450.3) = 26.9, *p* < 0.001
	Morning	57.1 ± 13.2	20.7 ± 10.9	33.5 ± 15.5	29.3 ± 16.1	F(1,30.8) = 2.6, *p* = 0.118	F(1,450.3) = 99.5, *p* < 0.001	F(1,450.3) = 67.4, *p* < 0.001
	Late evening	30.0 ± 14.1	28.0 ± 9.5	56.3 ± 7.5	32.4 ± 13.6	F(1,31.8) = 18.8, *p* < 0.001	F(1,450.7) = 47.6, *p* < 0.001	F(1,450.7) = 35.3, *p* < 0.001
Mean illuminance (log_10_ lux) ^1^	Daily	2.3 ± 0.3	2.2 ± 0.4	2.2 ± 0.4	2.2 ± 0.3	F(1,31.1) = 0.2, *p* = 0.68	F(1,450.3) = 3.7, *p* = 0.054	F(1,450.3) = 1.3, *p* = 0.248
	Morning	2.4 ± 0.4	1.9 ± 0.8	2.3 ± 0.5	1.9 ± 0.8	F(1,31.6) = 0.0, *p* = 0.949	F(1,429.3) = 24.9, *p* < 0.001	F(1,429.3) = 0.2, *p* = 0.659
	Late evening	0.8 ± 0.4	0.7 ± 0.3	1.3 ± 0.3	0.8 ± 0.6	F(1,31.5) = 5.9, *p* = 0.021	F(1,443.5) = 22.9, *p* < 0.001	F(1,443.5) = 14.1, *p* < 0.001
Frequency of alarm usage (%) ^2^		92.7	26.8	72.7	41.3	X2(1,481) = 8.5, *p* = 0.004	X2(1,480) = 94.6, *p* < 0.001	X2(1,479) = 19.5, *p* < 0.001

^1^ Data obtained from accelerometry recordings; ^2^ data obtained from sleep logs.

**Table 3:** Mixed-effects linear regressions for estimating sleep parameters (454 observations).

	Sleep Onset				Sleep End				Sleep Duration			
	b ± SE	t	df	*p*	b ± SE	t	df	*p*	b ± SE	t	df	*p*
Intercept	0.3 ± 0.2	1.2	70.1	0.241	8.8 ± 0.3	30.4	47.9	<0.001	8.5 ± 0.2	36.7	86.7	<0.001
Training shift (1 = Late evening)	1.1 ± 0.3	4.2	33.9	<0.001	1.3 ± 0.4	3.4	32.8	0.002	0.1 ± 0.3	0.4	35.4	0.709
Type of day (1 = Weekend day)	1.9 ± 0.2	12	434.5	<0.001	1.2 ± 0.1	8.1	429.2	<0.001	−0.7 ± 0.2	−4.2	437.9	<0.001
Alarm (1 = Yes)	0.1 ± 0.2	0.5	448.0	0.592	−1.6 ± 0.1	−11.3	436.9	<0.001	−1.6 ± 0.2	−9.8	452.3	< 0.001
Morning illuminance (log_10_ lux)	−0.3 ± 0.1	−4.8	453.9	<0.001	−0.1 ± 0.1	−2.1	445.2	0.038	0.2 ± 0.1	2.3	447.8	0.024
Late evening illuminance (log_10_ lux)	0.1 ± 0.1	2.1	452.8	0.036	0.2 ± 0.1	2.7	440.6	0.007	0.0 ± 0.1	0.5	453.5	0.589
Morning MVPA (minutes)	−0.0 ± 0.1	−0.5	454.0	0.631	−0.0 ± 0.1	−0.3	443.5	0.759	−0.0 ± 0.1	−0.1	450.7	0.890
Late evening MVPA (minutes)	0.4 ± 0.1	4.8	453.4	<0.001	0.1 ± 0.1	1.4	441.7	0.172	−0.3 ± 0.1	−3.2	452.9	0.001
Pseudo-R^2^ marginal/conditional	0.45/0.57				0.47/0.69				0.19/0.31			

Note: Time was converted to decimal hours, and sleep onset was additionally centered at midnight. Both minutes of MVPA and illuminance were mean-centered and standardized, so b should be interpreted as the change in the sleep variables associated with 1 standard deviation increase in these variables. MVPA: Moderate-to-vigorous physical activity; DF: degrees of freedom; SE: standard error.

## Data Availability

The data presented in this study are available on request from the corresponding author.

## Appendix A

**Table A1:** Mixed-effects linear regressions for estimating sleep parameters (454 observations).

	Sleep Onset				Sleep End				Sleep Duration			
	b ± SE	t	df	*p*	b ± SE	t	df	*p*	b ± SE	t	df	*p*
Intercept	0.7 ± 0.4	2.0	199.8	0.052	9.6 ± 0.4	25.3	113.4	<0.001	8.8 ± 0.4	24.6	242.5	<0.001
Training shift (1 = Late evening)	1.5 ± 0.3	5.2	31.4	<0.001	1.3 ± 0.4	3.6	31.1	<0.001	−0.1 ± 0.2	−0.6	30.6	0.575
Type of day (1 = Weekend day)	2.0 ± 0.2	12.6	461.1	<0.001	1.2 ± 0.1	8.5	456.4	<0.001	−0.8 ± 0.2	−4.7	464.4	<0.001
Alarm (1 = Yes)	0.0 ± 0.2	0.3	476.8	0.788	−1.7 ± 0.1	−12.3	465.7	<0.001	−1.7 ± 0.2	−10.3	482.3	<0.001
Daily illuminance (log_10_ lux)	−0.3 ± 0.1	−2.4	482.4	0.015	−0.3 ± 0.1	−3.3	471.8	0.001	−0.1 ± 0.1	−0.5	480.0	0.652
Daily MVPA (minutes)	0.0 ± 0.1	0.2	472.5	0.838	0.1 ± 0.1	1.0	481.1	0.328	0.0 ± 0.1	0.2	430.0	0.854
Pseudo-R^2^ marginal/conditional	0.38/0.52				0.46/0.69				0.18/0.30			

Note: Time was converted to decimal hours and sleep onset was additionally centered at midnight. Both minutes of MVPA and illuminance were mean-centered and standardized, so b should be interpreted as the change in the sleep variables associated with 1 standard deviation increase in these variables. MVPA: Moderate-to-vigorous physical activity; DF: degrees of freedom; SE: standard error.

**Table A2:** Mixed-effects linear regressions for estimating sleep parameters adjusted for the previous sleep end (421 observations).

	Sleep Onset				Sleep End				Sleep Duration			
	b ± SE	t	df	*p*	b ± SE	t	df	*p*	b ± SE	t	df	*p*
Intercept	0.3 ± 0.2	1.5	73.9	0.144	8.9 ± 0.3	31.3	49.2	<0.001	8.6 ± 0.2	36.1	90.1	<0.001
Training shift (1 = Late evening)	1.0 ± 0.3	3.9	33.1	<0.001	1.2 ± 0.4	3.4	32.5	0.002	0.0 ± 0.3	0.2	37.4	0.866
Type of day (1 = Weekend day)	1.8 ± 0.2	11.5	400.7	<0.001	1.1 ± 0.1	7.3	395.4	<0.001	−0.8 ± 0.2	−4.6	404.4	<0.001
Alarm (1 = Yes)	0.1 ± 0.2	0.7	414.9	0.489	−1.7 ± 0.1	−11.4	402.6	<0.001	−1.8 ± 0.2	−10.0	418.0	<0.001
Morning illuminance (log_10_ lux)	−0.3 ± 0.1	−4.4	420.9	<0.001	−0.1 ± 0.1	−1.2	410.8	0.218	0.2 ± 0.1	2.9	419.0	0.004
Late evening illuminance (log_10_ lux)	0.1 ± 0.1	1.7	421.0	0.087	0.1 ± 0.1	2.0	409.4	0.051	0.0 ± 0.1	0.4	419.5	0.658
Morning MVPA (minutes)	0.1 ± 0.1	0.7	410.1	0.477	−0.0 ± 0.1	−0.2	417.2	0.816	−0.0 ± 0.1	−0.5	399.7	0.640
Late evening MVPA (minutes)	0.4 ± 0.1	4.7	420.7	<0.001	0.1 ± 0.1	1.2	410.8	0.217	−0.3 ± 0.1	−3.1	418.1	0.002
Previous sleep end	0.3 ± 0.1	2.6	409.9	0.011	0.1 ± 0.1	1.4	417.0	0.158	0.0 ± 0.1	0.3	398.8	0.745
Pseudo-R^2^ marginal/conditional	0.47/0.57				0.48/0.69				0.21/0.32			

Note: Previous sleep end was included as control. Number of observations was smaller due to the absence of information on previous sleep end. Time was converted to decimal hours, and sleep onset was additionally centered at midnight. Minutes of MVPA, illuminance, and previous sleep end were mean-centered and standardized, so b should be interpreted as the change in the sleep variables associated with 1 standard deviation increase in these variables. MVPA: moderate-to-vigorous physical activity; DF: degrees of freedom; SE: standard error.
